# The Study of Medicine

**Published:** 1826-04

**Authors:** 


### VIII.

*The Study of Medicine. Second Edition.* By John Mason Good, M.D. F.R.S. F.R.S. E. Mem. Am. Phil. Soc. and F.L.S. of Philadelphia. 5 vols. 8vo, pp. lxv. 629, 662, 518, 688, 739. London, 1825, Baldwyn & Co.

A demand, thus early, for a second edition of the work before us, is a circumstance not less creditable to the discernment of the medical public, than it is flattering to the author; and it has afforded to Dr. Good, on opportunity of revising his labours; of which, as will be perceived, he has not failed to avail himself. The work has received an addition of about 300 pages, and is now distributed into five instead of four volumes.

Very many of the articles are extended, and, in our opinion, improved, by citations from the most eminent among the medical writers of Germany, France, and Italy. Several additional histories and illustrations have been furnished, by publications in our own country, of a very recent date; and on the subject of tropical diseases, information has been derived from some valuable, but hitherto unpublished, documents, addressed officially to the Army Medical Board.

In the advertisement prefixed to this edition, after a brief notice of its chief alterations and improvements, the author offers some apology for his infrequent reference to pathological anatomy; an omission which, we have reason to believe, some of his readers will regard as a defect. He urges, among other reasons, the often-repeated argument, that dissection exhibits, in a great many instances, rather the effect of disease, than the disease itself. But while we admit that this is true, we cannot give to it so much weight as the author seems to expect. Nor, in fact, does it appear to us to furnish the shadow of a reason for excluding pathological anatomy from the study of medicine, or even for assigning to it a subordinate place. The only way in which we are permitted to study *causes* is in their effects. Every morbid phenomenon, presented to the senses of the physician, and every morbid sensation described by the patient, is but an effect more or less removed from that primary disturbance of function, into which the imagination may, perhaps, resolve the commencement of all idiopathic disease; and in the entire course of almost every fatal malady, from the first functional deviation, to the final extinction of life, there is no effect so constant, and so definite, as derangement of structure; nor any one, which it so much interests the physician to ascertain, if possible, during the life of his patient. By the opinion he forms, as to the existence or non-existence of structural derangement; by his conclusions as to the nature, extent, and progress, of this derangement, will his prognosis, and his treatment be directed. Nor do we know any other means, by which the student can be so well educated to an accurate interpretation of symptoms, to the discrimination of morbid features, and the recognition of morbid processes, as by the constant habit of uniting in his mind, as much as is in his power, the intimations of disease which he has witnessed during the patient's life, with the changes which dissection exhibits after his death.

It is indeed undeniably true, that errors have been, and are daily committed in *post mortem* examinations; that incidental results are mistaken for those that are essential, and that appearances produced in the last struggles of expiring humanity, are confounded with those belonging to the primary malady. But what is this more than to say of morbid dissection, that which must be confessed and borne in mind at every step throughout the pursuit of medical truth, and of all other truth; that error constantly besets us; that the right path is single; but the wrong are innumerable; and that real knowledge is only to be obtained by a diligent, comprehensive, rigorous, examination and comparison of facts.

In the first volume, under the article Parabysma Hepaticum, the author has introduced some observations on the formation, and growth of tumours. After having remarked, that tumours sometimes consist of an enlargement, or extension of the general structure of the affected organ, and sometimes of distinct lumps or tubera of a very different structure, he goes on to observe:

"The simplest mode of conceiving of their origin is by the deposit of some living fluid into a cell of the cellular structure, or the follicular gland of a mucous membrane, possessing an increased excitement by the pressure of the surrounding parts, or from some other cause of irritation.

Mr. Hunter believed that 'the living fluid which has the greatest tendency to assume a vascular structure when thus collected or effused is the coagulable part of the blood,' for which opinion there seems to be a great reason: and hence those who have chiefly supported his doctrines in our own day, and especially Sir Everard Home and Mr. Abernethy, confine the origin of vascular tuberous growths to the sanguiferous system, and especially its coagulable part alone; while Dr. Baron has still more lately restricted them quite as absolutely to the absorbent system; contending that our hopes of being able to avert or cure such maladies must rest upon some other means than those which are merely calculated to subdue vascular action.

"Either of these views appears too narrow. Mr. Hunter has sufficiently shown, that a living principle appertains to almost all the fluids of the living body that are formed for its accretion, though the animal oil seems to possess less than any of the rest. He has shown it to exist in the chyle; it is known to every one to exist in the semen; and is transferred to the egg when it first drops from the body of the mother, and before a single particle of blood is elaborated. It is this, in truth, which is the instinctive principle of healthy living matter, whether animal or vegetable; instinct itself being nothing more than the simple law of life, or of such living principle in a state of activity or operation, directed to the definite end of completing single organs or the general system, preserving them in health, or reproducing them for future use.

"It is hence probable, that most, if not all, the living fluids, and not merely those of the coagulable part of the blood, or the semen, have a tendency to produce new forms and tissues, and will do so under a particular form of excitement, if duly supplied for that purpose. So long as a state of health, or the natural law of the instinctive principle influences them, these productions will be uniform and definite; but if the healthy power decline, and the natural form dependent on it cease, the action still continuing without a modifying guide, the productions must be indefinite and anomalous in every possible diversity, and hence alone, as it appears to me, can we account for the elabcration of all those infinite varieties of fluids or of fabrics which different tumours present to us."

The author has an evident partiality for this explanation; as he repeats it in very similar terms in the third volume, when treating of tubercular phthisis. But to what does it amount? A state of perfect health implies a perfect performance of all the functions of the animal. One of these functions is the formation of new parts: a healthy body forms healthy parts: an unhealthy body forms unhealthy parts. Does health, or the want of it in the animal which forms these parts, and in the parts themselves, merely imply the presence or the absence of this "modifying guide," this "simple law of life," this "instinct?" What is this instinct on which so much depends? Has it a separate existence? Is it a distinct principle; or is it a quality, resulting from certain conditions of animated bodies? How far does it differ from the modifying guide, which regulates the phenomena of galvanism, of magnetism, of elective attraction, and of mechanical impulse? New facts are always valuable. New theories are valuable in proportion to the facts they embrace and explain: new terms are always suspicious; but most of all, old terms in a new sense. The anima of Stahl; the archæus of Van Helmont; or the enormon of Hippocrates might, as we think, be substituted for the instinct of Dr. Good, not only without injury, but with manifest advantage.

In the second volume, besides a great many additions of considerable value, is a very well written chapter on that species of inflammation which arises from dissection, and which the author has named erythema anatomicum. Dr. Good regards this formidable complaint as referrible, not to simple irritation, in a constitution or idiosyncrasy of peculiar excitement; nor to the irritation of a putrescent fluid; but to inoculation of a specific virus; and this view of it is, he thinks, strongly confirmed by one fact, which he considers characteristic of the disease, namely, that the anatomic erythema commences with fever and constitutional disturbance, while the inflammation first shews itself about the shoulder or axilla: whereas, on the contrary, inflammation which arises from simple irritation, or from the irritation of putrescent animal matter, opens with local irritation at the point of the injury, and is generally an affection of a far less formidable character.

In this volume, at page 176, Dr. Good has been led, inadvertently, no doubt, into an erroneous statement, viz. that Sir Gilbert Blane had been *requested by the Admiralty* to examine and report upon the dreadful mortality which took place at the Island of Ascension, and on board the Bann, in the year 1823. This statement we can contradict in the most positive manner, and upon authority which will not be questioned. We should not have noticed it, did it not convey, by implication, an insult to the medical commissioners of the Navy, who certainly were the proper persons to be consulted by the Lords of the Admiralty on all such occasions—and are and were so consulted. The real circumstances of the case were these; Sir Gilbert Blane, seeing some notice of the disease in question in a newspaper, made an application to the Admiralty, to be allowed to peruse the official documents, and was properly referred to the Victualling Board. At this time, the only public document there gave very little information beyond the number of deaths; but, as Captain Phillips, the commander of the Bann, had returned to this country invalided, he, at Dr. Burnett's request, drew up a minute account of the affair, and on Sir Gilbert's calling at the office, Dr. B. at the request of Dr. Weir, put this document into his hands, with the official report, telling him at the same time, that he did not consider the information complete, and that the Medical Commissioners had written for more; intimating that they intended to make an official report to the Admiralty, as soon as their enquiry was finished. Without any further communication with Dr. Burnett, or in any shape requesting his permission to publish the information contained in a *private* communication with which he entrusted him, Sir Gilbert put forth a letter in the newspapers, addressed to the Board of Admiralty, and subsequently tried, by every means in his power, to have this document distributed to the Navy, which was, as *he knows,* not attended with success, to say the least of it.

Such are the facts—and such is the foundation for the *request of the Admiralty,* to investigate the subject of the fever at Ascension and in the Bann!

In the third volume, there are very many important additions, some of which we shall notice, but cannot enter into an extended examination of their merits.

Under the head of small-pox, the history of that disease is presented in a very enlarged form: and the questions of its origin and antiquity, together with the speculations of Dr. Willan, who contends for its identity with one of the varieties of plague, or λοιμος of ancient authors, pass in review.

Under the genus marasmus, is given the history of the disease, here termed Marasmus Anhæmia, or Exsanguinity; of which an example by Mr. Combe is contained in the Edinburgh Medico Chirurgical Transactions; and which disease prevailed as an endemic among the labourers, in a coal mine at Anzain, as described by Professor Halle in M. Corvisart's Journal.

In treating of phthisis, the author adverts to the use of the stethoscope. We hail every effort to extend the knowledge and the employment of this invaluable instrument. In the diagnosis of internal diseases, particularly of the thorax, the use of percussion and auscultation appears to us one of the greatest improvements in clinical practice which this age has produced; and however long indolence and prejudice may prevail against it, we rely with confidence that ultimately it will be generally adopted in this country.

Among the cachexies, Dr. Good has now introduced, for the first time, the genus melanosis. His account of it is taken from M. M. Breschet and Laennec, and also from a paper in the Edinburgh Medico-chirurgical Transactions.

On the subject of lues, the second edition varies very widely from the first, as will be seen by the following short extracts from each. In the first edition we read as follows. "It was regarded by Mr. Hunter, as a pathognomonic character of syphilis, first, that it never ceases spontaneously: secondly, that it is uniform and progressive in its symptoms: thirdly, that it is only to be cured by mercury: how far the last position may admit of such a modification as may leave it open to occasional exception in other climates, it is not worth while to enquire. In our own, the whole of these dicta stand upon a rock of facts progressively accumulating and confirmed by every day's experience."—*Edition of* 1822, Vol. ii. p. 847.

But, however such doctrines at that period might have surprised us from the learned and intelligent author of these volumes, we feel that Dr. Good is entitled to the praise of candour in his recantation: for in the present edition, after reciting these dicta of Mr. Hunter, he adds: "Sufficient proof has been offered, that not one of these points holds good, without a considerable degree of modification, whether in respect to the primary, or the secondary symptoms of these maladies."

If, in the history of medicine, there be one fact fitted above every other, to teach modesty and distrust of themselves to those who profess to teach it, it is most unquestionably to be found in the subject we are now contemplating. Among all who have attempted in modern times, to explore the laws which regulate animal life in health, and in disease, no one has been more zealous, more indefatigable, more sagacious, and we must add, more successful than John Hunter. One of ten axioms he deduced, from all the immense mass of his observation and experience, is, that syphilis is incurable without mercury. For thirty years, there was scarcely a surgeon in the empire, who dared to doubt the truth of this assertion; every patient who escaped from lues, with his life, and his nose, was regarded as affording additional testimony to the power of this specific; and medicine triumphed in her undisputed and undivided sovereignty over this loathsome disease: some puzzling facts were from time to time, placed before the public; but as the worshippers of Galen, deeming it impossible for him to err, contended that the human structure must have undergone some mighty alterations since he described it; so the disciples of Hunter imputed anomalies without end to Nature, rather than depart from the doctrines of their preceptor. But, at length, authority is compelled to bow before the irrefragable evidence resulting from accurate and extended observation. That alleged rock of facts which was confirmed, as Dr. Good had told us, and as the world generally believed, by every day's experience proves, but like those rocks of cloud which sometimes deceive the unpractised mariner; and at this day there are hundreds and thousands of living witnesses who can attest from their own personal experience, that syphilis is, at least in a very large proportion of cases, not only curable, but has been actually and permanently cured by the unaided powers of Nature.

In the fourth volume is contained a very well written and valuable article on muscular distortion of the spine; in which the lateral and the angular curvature are well described, and contrasted; the merely muscular affection is distinguished from the osseous, and the opinion of those who regard both as of strumous origin is ably combated.

In the fifth volume we remark, among other improvements, that the chapter on dropsy is considerably enlarged, and is somewhat more in accordance than before with the opinions which at present prevail on that subject. Still, the author regards dropsy as being, in most instances, a disease of constitutional debility. In a work like the present, we cannot but lament such vague, and we had almost said unmeaning pathology. Constitutional debility may be a very convenient term with the teachers of popular medicine, but we are at a loss to conceive how it has become such a favourite with Dr. Good. One of the most usual and well-known effects of disease, is a diminution of the mechanical force with which the voluntary muscles contract. When, to denote this effect, the term debility is used, it is perfectly intelligible. If the same term be employed to denote certain changes produced by disease in the functions of circulation and respiration, its propriety becomes at least doubtful; when applied in reference to certain supposed conditions of the functions of sensation, of secretion, of absorption, of assimilation, it is nearly unintelligible. But when, instead of denoting an effect of disease in one or more of the functions, it is extended to the whole system, and made to designate a supposed cause of disease, we wish that obscurity were the only fault of such language; we believe it to be highly mischievous.

P. S. The above short criticism on Dr. Good's new and amended edition, was written by a gentleman of talent and observation, who is not personally known to Dr. Good, and who resides far distant from the metropolis. But the Editor of this Journal cannot avoid adding a few remarks on some animadversions which Dr. Good has made on him in the second volume of the work, p. 554, *et seq*. Dr. Johnson must, of course, feel grateful to the learned author of the Study of Medicine, for the flattering expressions with which he prefaces his remarks; but he confesses that he would have been as well pleased with less commendation, and a more correct representation of his sentiments.

"Some writers," (says Dr. Good,) "however, as Piso formerly, and Dr. James Johnson in our own day, have carried this view of the subject (dysentery as connected with hepatic derangement) considerably farther than my late learned and venerated friend Dr. Chisholm ever intended; for they have boldly reversed the general opinion that has prevailed, and especially since the days of Sydenham, and contended that the *liver* itself forms, in every instance, *the primary seat* of the disease, the intestines being only affected secondarily." *Vol.* ii. *p.* 554.

Now Dr. Johnson appeals to the public, nay to Dr. Good himself, whether the above be not a complete misapprehension, to say the least, of his sentiments.

"In every case of dysentery," (says Dr. J.) "that has come within the range of my observation (and the number has not been inconsiderable) *two functions* were invariably disordered from the very onset, and soon drew other derangements in their train. These were, the functions of the *skin* and of the liver, or *perspiration* and biliary secretion.

"These then are the *two* first links of that morbid chain which *connects the remote cause with the ostensible form of the disease*." * * * "Some other invisible, or at least very obscure links, are now to be noticed:—for however confidently a *proximate cause* may be decided on in colleges and closets, it is, in Nature, a *series of causes*. The equilibrium of the circulation and excitability becomes disturbed. In consequence of the torpor in the extreme vessels on the surface, the volume of blood is directed to the interior, and the balance is still farther broken by the check which the portal current meets in the liver, from a corresponding torpor in the extreme or secreting vessels of that organ; the effect of which is, that the plethora in the cœliac and mesenteric circles is now greatly augmented, and febrile symptoms commence. The perspiration being stopped, a vicarious discharge of mucus and acrid serum is thrown from the extremities of the turgid mesenteric vessels upon the internal surface of the intestines, which by this time are in a state of irritability. The disease now begins to exhibit itself unequivocally, by the uneasiness in the bowels, the frequent desire to stool, and the mucous discharges. We may now plainly perceive how all those consequences, which have so often passed for causes, can arise. If the plethora be great, blood itself will be poured out from the mouths of the distended mesenteric and meseraic vessels; hence, inflammation and ulceration may ensue. If any hardened fæces lurk in the cells of the colon, they will be grasped by the irritable circular fibres of the intestines, and rings or strictures will augment the tormina and griping in the bowels."—*Tropical Climates, 3d Edition*, p. 194.

Now, with what degree of reason can Dr. Good make Dr. Johnson to state that the primary seat of dysentery is invariably in the *liver*, after reading the above passages? In the first place, Dr. J. disclaims the idea of there being any such thing as *a proximate cause* in the disease at all—and, in the second place, he only states the *function of the liver* to be disturbed, in common with that of the surface, as a link in the chain "which connects the remote cause with the ostensible form of the disease." Here then is the head and front of Dr. Johnson's offending; and yet, with singular inconsistency, if not shortness of memory, Dr. Good, in the very page preceding that wherein he makes the charge against Dr. Johnson, affirms himself, that "the functions of the skin and of the liver are, *from the very first*, as well as throughout the whole course of the disease, considerably disturbed, &c." *Vol.* ii. *p.* 553.

After this, it is quite needless to make any further defence. Dr. Good has set up a man of straw, of his own creation, to be knocked down—but with what degree of credit to himself, Dr. Johnson leaves to the public, and to the learned author to estimate.

In the same volume, Dr. Good has taken upon himself to decide on the *practice* which Dr. Johnson adopted in Tropical Climates. But Dr. J. cannot admit Dr. Good to be the best judge on that point. Dr. G. has neither seen nor felt the disease, and, therefore, his knowledge is taken entirely from books. He says, that had Dr. Johnson waited a few months, (for the publications on fever and dysentery in Ireland) he would have found not only his opinions respecting the non-contagion of tropical dysentery, but his hepatic theory, and mercurial practice completely unhinged. Now, as Dr. Johnson did not construct his creed or his practice on the latest book which he might happen to have read on the particular subject, but on a careful observation of facts and phenomena at the bed-side of sickness, so he does not deem it necessary to change them with every edition of his work. And least of all, would he deem it incumbent on him to cancel the opinions he had formed respecting the contagion, the pathology, or the treatment of tropical idiopathic dysentery, occurring among robust Europeans in the Indies, because they did not quadrate with those of Dr. Cheyne, when treating of a low fever prevailing among the half-starved rabble of Ireland, where the dysenteric affection was a mere feature or occasional form of the reigning epidemic! How Dr. Good could dream of setting up a parallel between diseases and circumstances so essentially different, is really not easy to imagine. Dr. Johnson has moreover to state that, so far from cancelling his opinions, in a new edition of his work now in the press, an extensive communication with practitioners in both hemispheres, has amply confirmed what was first founded on actual experience. Such is the difference between the creed formed in the closet from books, and the conviction resulting from personal observation at the bed-side of sickness.

Lastly, Dr. Johnson is accused of *scoffing* at Sydenham, in consequence of a passage in the Medico-Chirurgical Review, wherein the writer avers, that there is no proof of any *post mortem* investigations in the works of the English Hippocrates. If Dr. Good had given himself time for a moment's reflexion, his natural good sense, or even a sense of common justice, would have suggested to him the impropriety of attaching every sentiment in a medical review to the managing editor. Dr. Johnson, however, disdains to use a subterfuge, and avers, without fear of contradiction, that there is not a single fact in the writings of Sydenham, which would lead one to suppose he ever examined a dead body in dysentery, or any other disease. Sydenham has always been considered and revered by Dr. Johnson, as a careful observer of the phenomena of diseases in the living body, (a credit which is by no means due to many of his idolatrous admirers) but as to *pathology*, the Hippocrates of England was *much more ignorant* than the Sage of Cos.

With these few remarks in vindication of himself from a great misapprehension of his sentiments, Dr. Johnson concludes this article—assuring Dr. Good, that no man entertains a higher respect than he does for the learning and talents of the author of the "Study of Medicine." Dr. J. may perhaps be permitted to take this opportunity of expressing his conviction, that this second edition is incomparably superior to the first, and, that if it was the best book of the kind in its pristine form, it is now still more deserving of commendation and public patronage.

Dr. Johnson begs leave, in conclusion, to make known his intention of instituting a series of commentaries, in a succession of articles in this Journal, on the various diseases embraced in Dr. Good's work; hoping, should life and health be spared, to clear away some of the difficulties which beset the young practitioner, and lay before him a succinct, yet conspicuous view of the actual amount of our knowledge in the etiology, pathology, and treatment of diseases.

